# The influence of oral cavity physiological parameters: temperature, pH, and swelling on the performance of denture adhesives - in vitro study

**DOI:** 10.1186/s12903-024-03967-7

**Published:** 2024-02-09

**Authors:** Josephine Koehler, Anantha Narayanan Ramakrishnan, Christopher Ludtka, Jeremias Hey, Andreas Kiesow, Stefan Schwan

**Affiliations:** 1 Department of Biological and Macromolecular Materials, Fraunhofer Institute for Microstructure of Materials and Systems IMWS, Walter-Huelse Str. 1, 06120 Halle (Saale), Germany; 2 Department of Operative Dentistry and Periodontology, Martin-Luther-University Halle-Wittenberg, 06112 Halle, Germany; 3 Department of Engineering and Natural Sciences, Hochschule Merseburg, University of Applied Sciences, Merseburg, Germany; 4 Institute for Modelling and Simulation of Biomechanical Systems, Faculty of Civil and Environmental Engineering, University of Stuttgart, Stuttgart, Germany; 5 J. Crayton Pruitt Family Department of Biomedical Engineering, University of Florida, Gainesville, USA; 6 Department of Prosthetic Dentistry, University School of Dental Medicine, Martin Luther University Halle-Wittenberg, 06112 Halle, Germany

**Keywords:** Denture adhesive, Physiological parameters, Denture, Rheology, Viscoelastic, Swelling, Artificial Saliva

## Abstract

**Background:**

The various physical and chemical conditions within the oral cavity are hypothesized to have a significant influence on the behavior of denture adhesives and therefore the overall comfort of denture wearers. As such, this study aims to understand the influence of oral cavity physiological parameters such as temperature (17 to 52 °C), pH (2, 7, 10), and denture adhesive swelling due to saliva (20–120%) on the behavior of denture adhesives. This study further aims to emphasize the need for a collective approach to modelling the in-situ behavior of denture adhesives.

**Methods:**

Rheological measurements were carried out using the Super Polygrip Ultra fresh brand denture adhesive cream to evaluate its storage modulus (G´) and loss modulus (G´´) values at a range of physiologically relevant temperatures, pH values, and degrees of swelling, to represent and characterize the wide variety of conditions that occur within the oral cavity.

**Results:**

Rheological data was recorded with respect to variation of temperature, pH, and swelling. Overall, it can be seen that the physiological conditions of the oral cavity have an influence on the rheological properties of the denture adhesive cream. Specifically, our data indicates that the adhesive’s mechanical properties are weakly influenced by pH, but do change with respect to the temperature in the oral cavity and the swelling rate of the adhesive.

**Conclusions:**

Our results suggest that the collective inter-play of the parameters pH, temperature and swelling ratio have an influence on the behavior of the denture adhesive. The results clearly highlight the need for developing a multi-parameter viscoelastic material model to understand the collective influence of physiological parameters on the performance of denture adhesives. Multi-parameter models can also potentially be utilized in numerically simulating denture adhesives using finite element simulations.

## Background

Both partial and complete dentures are classic treatments for restoring functionality and aesthetic appearance in cases with the loss of several or all teeth. Patients with these prosthetics often also use denture adhesive cream [1]. The use of adhesives has been reported to make patients feel more secure in their social environment, being less afraid that the denture will loosen or fall out when they speak or eat [2–6]. Dentists naturally strive for ideal retention when fabricating a denture, which do not necessarily require the use of an adhesive cream. Nevertheless, many patients use denture adhesives. In addition to its positive effect on patient satisfaction, studies have also shown that denture adhesives significantly improve retention, stability, and chewing performance [2, 3, 6–9]. This applies to both well-fitting and ill-fitting dentures. However, an ill-fitting denture can lead to soft tissue trauma (pressure points, etc.) and accelerated alveolar ridge atrophy. Thus, denture adhesives on their own cannot replace relining by the dentist [3, 4, 10]. In its mode of operation, the denture adhesive supports the retention mechanisms of a denture by increasing the viscosity of the saliva between the denture and the supporting mucosa [5, 7]. Responsible for this are the defined “active ingredients” of an adhesive, such as karaya gum, sodium carboxymethylcellulose, and synthetic polymers (e.g. polyethylene oxide, acrylamides, acetic polyvinyl), which swell and become more viscous in the presence of saliva [5]. In addition, “inactive ingredients” such as petrolatum, mineral oil, and polyethylene oxide act as binders in these products [11]. Since the ideal bonding agent should ensure retention of the denture for 12 to 16 h [5], the product should be exposed to the conditions in the oral cavity for a longer period of time for relevant testing.

Due to the presence of saliva, the oral cavity is always a moist environment under normal physiology conditions. Lenz et al. (2000) describe the oral cavity and oropharynx regarding its composition of different cell types and tissues, including salivary gland epithelia [12]. Saliva is produced by these major salivary glands (parotid gland, sublingual gland, and sublingual gland) and several minor salivary glands scattered throughout the oral cavity [13]. Saliva has a key role in chewing, swallowing, and digestion, as well as maintaining moisture in the oral cavity [13]. Consequently, denture adhesives are also influenced by the presence and quantity of saliva [14].

As another consideration, saliva has a certain pH value, which averages 6.8 in the unstimulated state and 7.8 in the stimulated state [15]. Factors such as the amount of salivary secretion, saliva mineral concentration, breathing through the nose versus the mouth, and diet have an influence on fluctuations in pH [15, 16]. The pH experienced in the oral cavity can be influenced by the type of food ingested, with values ranging from 1.0 to 10.5 being reported as a function of different foods [15]. According to the work of Fallahi et al. (2017) [17], under acidic conditions higher hydrogen bonding is observed between the polymer chains of denture adhesives. Meanwhile in the case of an alkaline environment, ionic bonding was potentially observed between the polymer chains, which lowers the adhesive strength of such denture adhesives.

Additionally, denture adhesives in the oral cavity are also exposed to changes in temperature. On average, a temperature of approximately 34 °C can be measured over a period of 24 h [16]. Temperature, like pH, is also subject to natural fluctuations caused by, among other things: food and beverage composition, changes in ambient temperature, breathing with the mouth closed or open, or smoking [18]. For example, temperatures ranging from 1.62 to 65.43 °C could be measured during consumption of hot or cold beverages in a study by Barclays et al. (2005) [19]. Although adhesive behavior is strongly influenced by physiological conditions, it is critical that its mechanical behavior is stable and maintained in regards to the wider range of potential temperature, pH, and water content [9, 20]. Gill et al. 2017 [21] test an adhesive formulation with two primary variants (hydrated and not hydrated), demonstrating the impact of swelling of the adhesive. Furthermore, Fallahi et al. (2017) [17] primarily investigate the denture adhesive at three specific temperatures in the oral cavity, i.e. 0, 37 and 60 °C. As the parameters in the oral cavity are changing dynamically, there is a need to understand the influence of even minor changes in temperature, pH, and other oral environment factors. As such, there is a need for an in-depth analysis of these parameters on the mechanical behavior of the adhesive formulation. To the author’s knowledge, past studies have not focused on these complex relationships and in many cases, they were not considered in great detail.

The aim of this study is to investigate the influence of oral cavity physiological parameters such as temperature, pH, and saliva-induced swelling on the mechanical properties of a denture adhesive cream. This study further aims to characterize this behavior through rheological studies.

## Methods

**Fig. 1:**
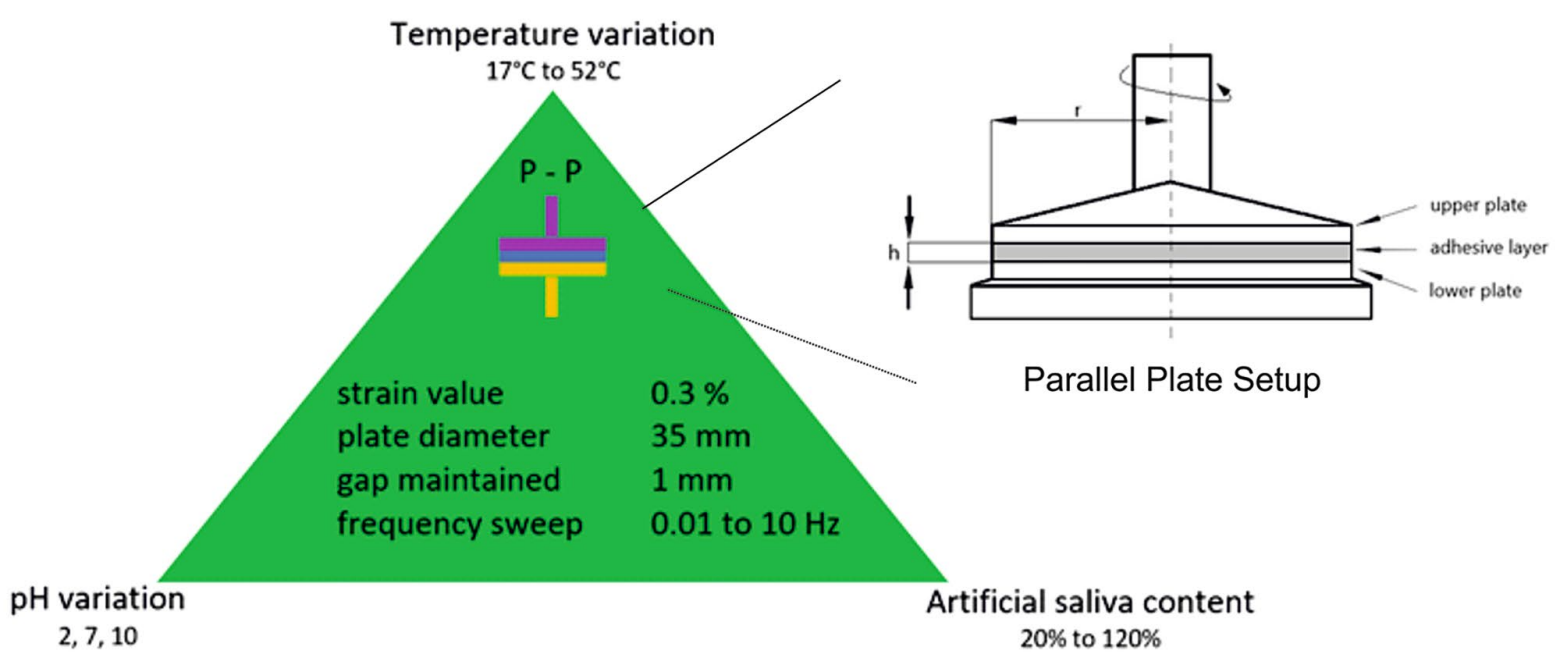
Determination of the viscoelastic behavior for the physiological chewing range by means of frequency sweep (0.01 to 10 Hz) test on a plate rheometer in the plate-plate (P-P) measurement configuration with a plate distance of 1 mm

An in vitro study of denture adhesive cream was conducted to determine the storage modulus, G´, and the loss modulus, G´´, to characterize the adhesive’s viscoelastic properties. To simulate the physiological conditions of the oral cavity for this purpose, three major parameters were considered. The factors evaluated are illustrated in Fig. 1. The pH of the artificial saliva was adjusted to acidic value of 2 and an alkaline value of 10, in addition to a neutral value of 7. For each of these values, the swelling rates of the adhesive cream were determined in order to simulate adhesives under the influence of swelling due to saliva.

### Denture adhesive and artificial saliva

Super Poligrip Ultra Fresh (GlaxoSmithKline, United Kingdom) denture adhesive cream, which is representative of the cream-type adhesives, was used for testing. Products of the same batch were used for all samples. Preparation of the artificial saliva was based on Pratten et al. (1998) [22]. The following ingredients were added to distilled water: 2.5 g/L hog gastric mucin, 3.5 g/L sodium chloride, 0.2 g/L calcium chloride, 0.2 g/L potassium chloride, 1 g/L ‘Lab-lemco’ powder, 2 g/L yeast extract and 5 g/L proteose peptone (all chemicals were supplied by Carl Roth, Germany). After autoclaving at 121 °C for 15 min, 1.25 ml of 40% urea was added to the artificial saliva. Measurements were performed with artificial saliva of different pH values. For this purpose, a pH meter with accuracy to nearest hundredth (S40-SevenMulti™ pH-Meter, Mettler Toledo), 10% NaOH solution, and 20% HCl solution were used to adjust the pH to 2, 7, or 10, respectively.

### Swelling ratio vs. time measurement

During preparation, the swelling rates of Super Poligrip® Ultra Fresh denture adhesive were determined for each pH value. The mass increase of the adhesive cream was determined after incubation in artificial saliva over a period of 2.5 h. For this purpose, 1.57 g of the adhesive cream was applied to individual, specially prepared cell sieves, the bottoms of which were replaced by a round piece of filter paper. The mass was determined every 10 min to the nearest thousandth using a precision balance. A total of 18 samples were analyzed to determine the swelling, with six samples for each of pH 2, 7, and 10, which took place at a constant temperature of 22 °C. With the subsequently calculated average values, the swelling rate per time could be determined. Using the software OriginPro 2019 (OriginLab Corporation, MA United States), graphs of these values could be generated and the time an adhesive sample has to rest in saliva to reach a certain swelling percentage could be calculated. Equation 1 was used for calculating the swell values.

1 $$\left(\frac{{m-m}_{0}}{{m}_{0}}\right)\text{*}100$$

### Rheological measurements

A rotational rheometer from the Haake company (Thermo Fisher Scientific Inc, MA United States) was used to quantify viscoelastic properties. The setup includes two congruent round metal plates (35 mm diameter), which are parallel to each other at a distance of 1 mm during the measurement. Parallel plate geometry was selected due to consideration of the higher viscosity of the denture adhesive being investigated. In addition, a shear rate of 0.3% and frequency range of 0.01 to 10 Hz were used. For each decade, 10 measurement points were determined, with three measurements being taken for each point. The average values of these were used for subsequent calculations. A total of 136 samples were measured, distributed as follows: across three pH values (pH 2, 7, and 10), eight temperatures (ranging from 17 to 52 °C, in 5 °C steps each) and on five to six swelling rates, which were determined depending on the respective pH value (see above). The desired temperature was set on the rheometer before each measurement.

To negate the potentially confounding effect of shear deformation, a new sample was prepared for each measurement. To prepare each measurement, a sample of the adhesive cream was placed in artificial saliva for an amount of time according to the data obtained in the swelling test. For this purpose, the sample was placed on a sample container with a bottom made of filter paper and placed in a 6-well plate containing artificial saliva (at pH 2, 7, or 10). After the time to reach the desired swelling rate had elapsed, the sample was removed and briefly placed on cellulose paper to allow excess saliva to drain. The prepared sample was then applied to the lower plate of the rheometer, and the measuring gap of 1 mm was subsequently approached. The excess sample was removed with the aid of a spatula and discarded.

The rheological experiments yielded values for the storage modulus (G´) and loss modulus (G´´) of the adhesive sample as a function of the applied strain and the initial measurement parameters (i.e. temperature, degree of swelling, and pH of the solution). These values were used to describe the viscoelastic material behavior of the denture adhesive.

### Statistics

In determining the threshold ratio, it was assumed that a normal distribution exists for each threshold level. Thus, the number of parallel samples for determination was set at 6 and the hypothesis was tested with the one-sample t-test. Since the starting material is all from the same lot (i.e. a homogeneous composition over the entirety can be assumed), the rheometric tests were carried out on one sample per test point. The significance of individual input parameters on the resulting relaxation spectrum for the output variables of storage and loss moduli were analyzed using the multiple linear regression analysis technique, which is a commonly used predictive analysis to examine whether a set of predictor variables can accurately predict the output variable. It was performed considering the entire domain of the three input variables: pH, swelling, and temperature, with a confidence level of 98%. The probability of seeing a response described by the *p*-value was compared with the significance level of 0.02, which is based on the selected confidence level of 98%. Initially a null hypothesis that the predictor variables of temperature, pH, and swelling ratio do not have an influence on the response variables is assumed and this is evaluated based on the evaluated *p*-values.

## Results

**Fig. 2:**
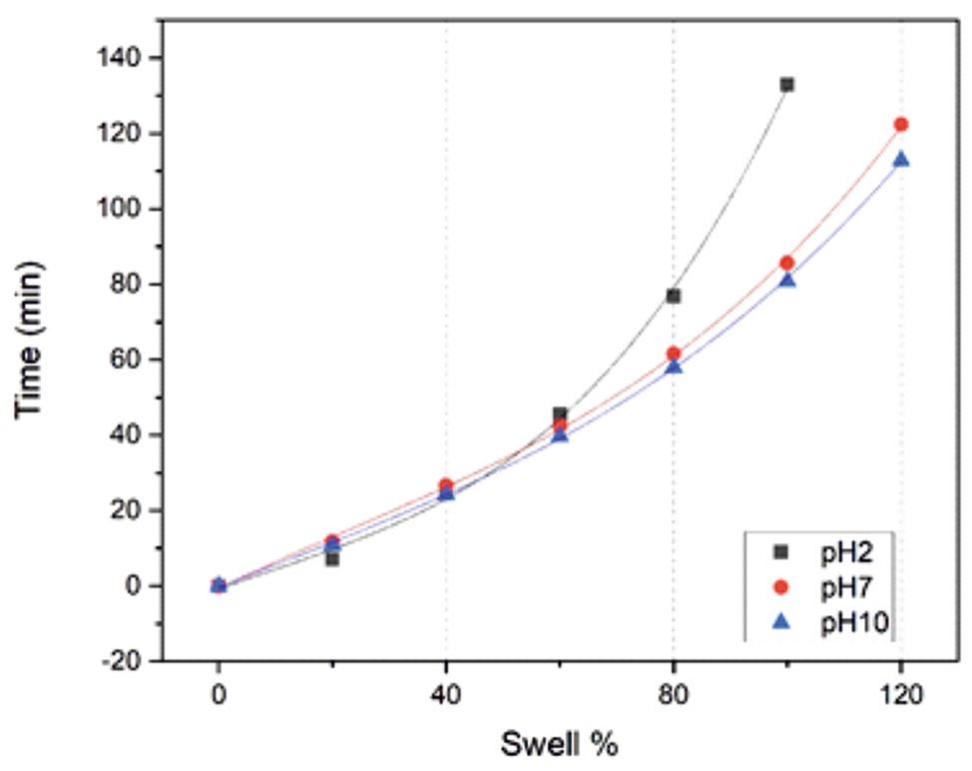
Time elapsed to attain a specific swelling percent ratio, for different pH values at which the adhesive was maintained

Based on the weight measurements for the adhesive creams at different time intervals, the time taken to attain a particular swelling percent at constant temperature was evaluated. This data is recorded in Table 1. This comparison for pH values of 2, 7, and 10 is further highlighted in Fig. 2. Beyond 140 min all the specimens approach saturation levels, i.e. the subsequent increase in swelling is infinitesimal with respect to time.

**Table 1:** Time in which the sample reaches a determined swelling percent, for different pH values

Room temperature (23 °C)	pH 2	pH 7	pH 10
Swelling (%)	time (min)	time (min)	time (min)
20	7.1	11.7	10.8
40	25.2	26.6	24.3
60	45.5	42.4	39.8
80	76.9	61.6	58.0
100	132.9	85.6	80.8
120	---	122.4	112.9

### Storage and loss modulus

**Fig. 3:**
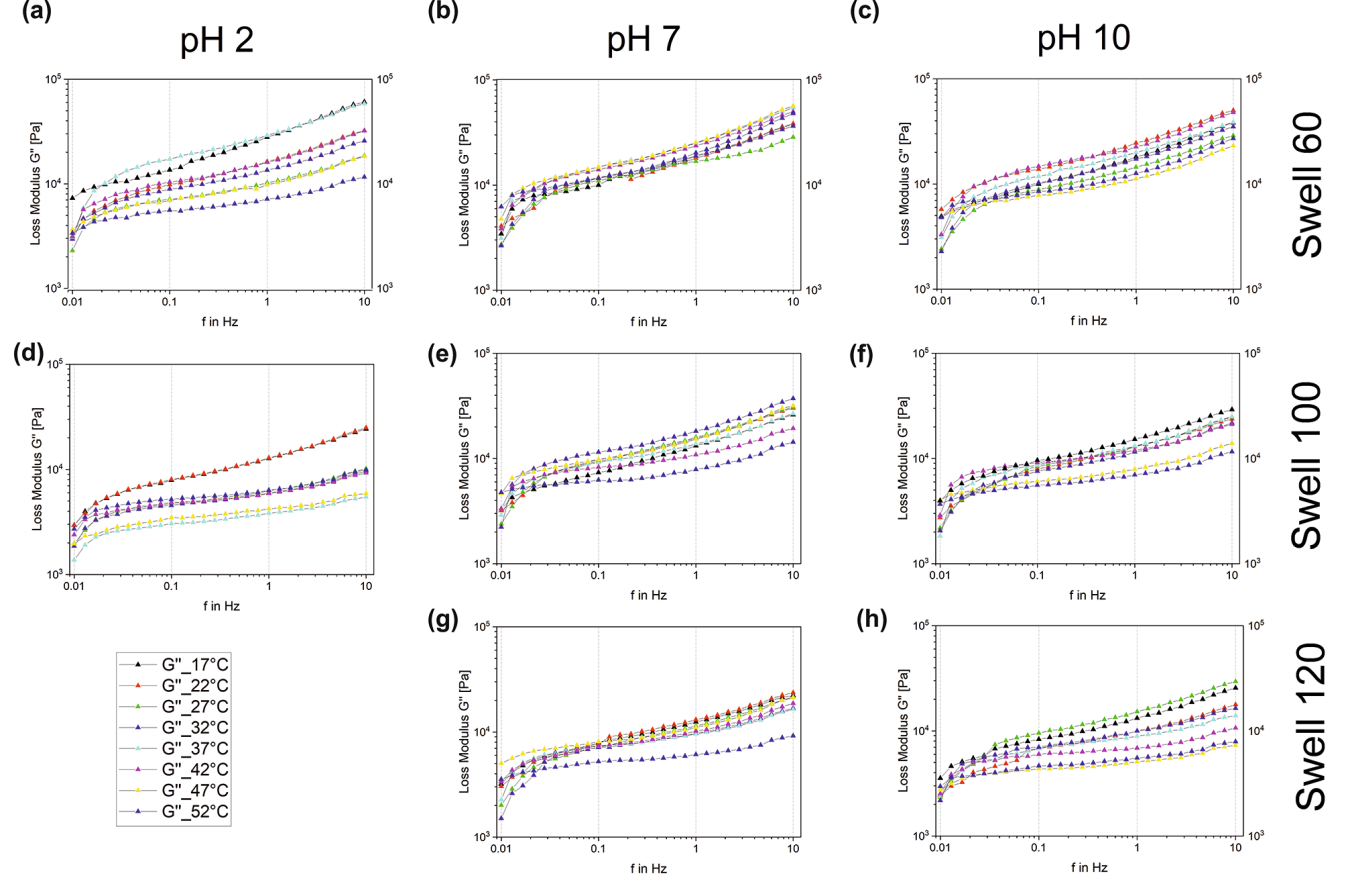
Plot of G´´ in the frequency range from 0.01 to 10 Hz at (**a**) pH 2 and swelling 60%; (**b**) pH 7 and swelling 60%; (**c**) pH 10 and swelling 60%; (**d**) pH 2 and swelling 100%; (**e**) pH 7 and swelling 100%; (**f**) pH 10 and swelling 100%; (**g**) pH 7 and swelling 120% and (**h**) pH 10 and swelling 120%

**Fig. 4:**
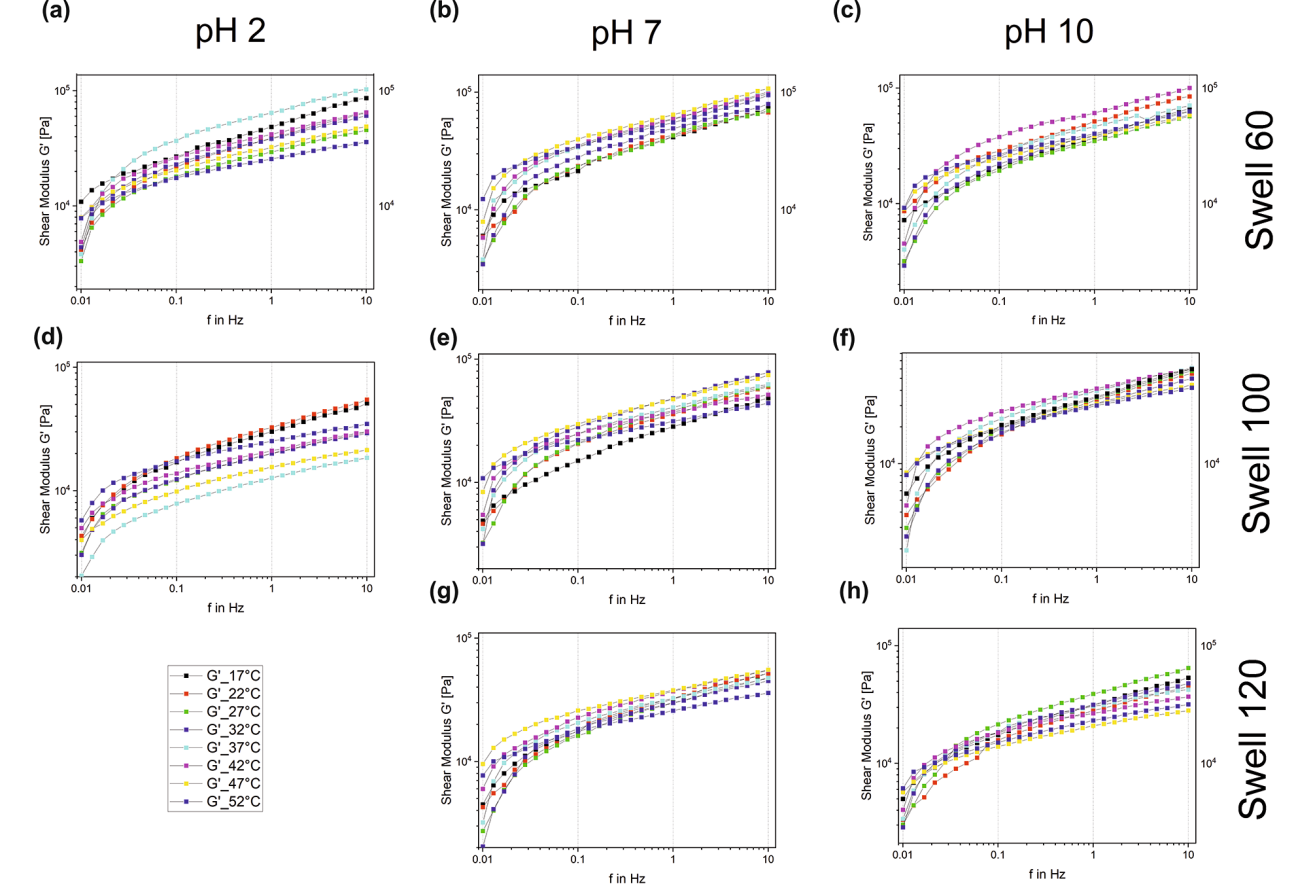
Plot of G´ in the frequency range from 0.01 to 10 Hz at (**a**) pH 2 and swelling 60%; (**b**) pH 7 and swelling 60%; (**c**) pH 10 and swelling 60%; (**d**) pH 2 and swelling 100%; (**e**) pH 7 and swelling 100%; (**f**) pH 10 and swelling 100%; (**g**) pH 7 and swelling 120% and (**h**) pH 10 and swelling 120%

The graphs in Figs. 3 and 4 show the changes in storage modulus, G´, and loss modulus, G´´, in the corresponding frequency range at different temperatures. The columns divide the graphs according to the pH of the artificial saliva in which the samples were swollen: on the left is for a pH value of 2, in the middle pH 7, and on the right pH 10. The rows show the values of equal swelling rate. The first line shows the values at 60% swelling, the second at 100%, and the third at 120%. As a swell rate of 100% for Polygrip at pH 2 is equal to the maximum value or near saturation of the sample, a lower plot in left column is omitted.

In general, it was observed that with increasing frequency, the values of G´ and G´´ also increased. The curves showed similar trends in all diagrams, although in the range from 0.01 to approximately 0.03 Hz a steeper slope of the curves was usually striking. In Fig. 3 (a) the values of different temperatures for pH 2 and swelling 60% are shown. Here it was noticed that the curves for 17 °C, 47 °C, and 52 °C showed approximately linear progressions. As the frequency increased, the fluctuations in the values for G´´ at different temperatures became larger; the curves fanned out further. The highest values occurred at a temperature of 32 °C, and the lowest at 52 °C. The values of 22 and 52 °C stood out in Fig. 3 (b) because of their weaker and almost constant increase. In general, the curves of different temperatures in this diagram are close to each other and the values hardly vary. The values here are largest in the temperature range between 37 and 47 °C, and smallest at 27 °C. Figure 3 (c) represents the measurement results of different temperatures for pH 10 and swelling 60%. Here it was noticed that the relative variation in G´´ values at different temperatures was negligible as frequency was varied between 0.01 and 10 Hz, i.e. the values hardly fluctuated. At a temperature of 47 °C, G´´ was at its lowest. For this, an approximately linear monotonic behavior was again noticed, which is also true for 17 and 52 °C. Figure 3 (d) shows similarities to Fig. 3 (a). The values for G´´ again varied more for the different temperatures. This also increased with increasing frequency. Under colder measurement conditions, higher G´´ values could be seen. Figure 3 (e) also demonstrates that the span of G´´ values for different temperatures increased with increasing frequency. This is also true for Fig. 3 (f) and Fig. 3 (g). In these two diagrams, except for the curve for 52 °C and in Fig. 3 (f) that for 47 °C, hardly any fluctuations occurred for G´´ at different temperatures. On the contrary, the curves in Fig. 3 (h) again show larger temperature-dependent changes observed with increasing frequency. It was noticed that in the low frequency ranges, the G´´ values are higher at higher temperatures, as seen for pH 7 and pH 10. With increasing frequency, this changed, and the colder temperatures showed higher values. The plots shown in Fig. 4 are very similar to those in Fig. 3 and differ only in the range of values of the results for G´ and are larger than that for G´´.

**Fig. 5:**
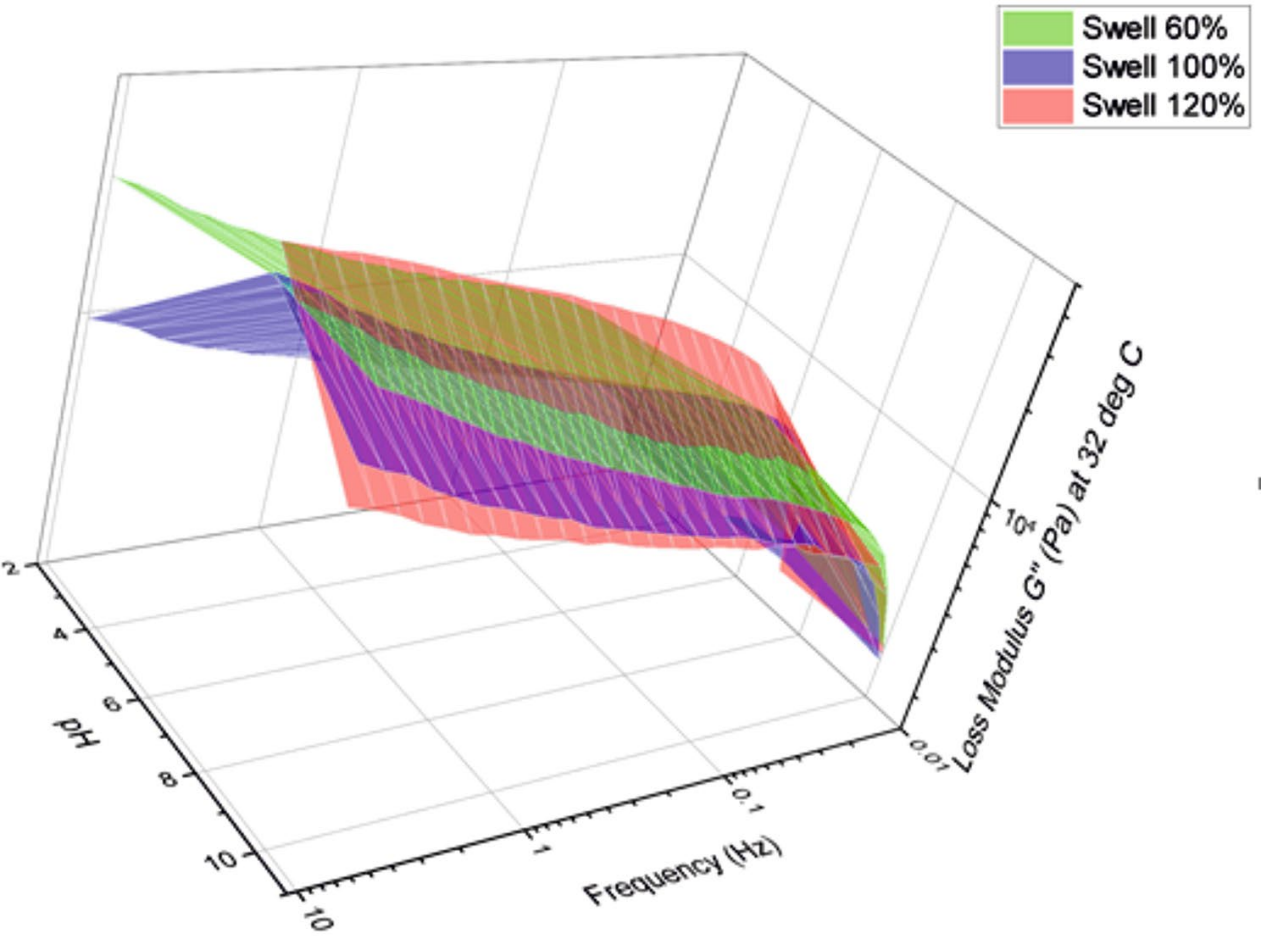
Loss modulus behavior of the adhesive cream with respect to both pH and frequency at a constant temperature of 32 °C and for varying swelling ratios from 60–120%

The 3D plot in Fig. 5 attempts to capture this combined influence of swelling and pH on the adhesive cream’s loss modulus behavior. This plot is for 32 °C, which was the closest measurement point to the described average temperature of 34 °C. The adhesive generally shows a higher loss modulus at pH 7 for all three swelling ratios depicted in Fig. 5.

The results obtained for the multiple linear regression test for the prediction of storage modulus is presented in the Table 2. The *p*-values for all three parameters of temperature, pH, and swelling were observed to be lower than the significance value of 0.02 for the storage modulus values. In other words, the storage modulus values can be estimated with the confidence level of the prediction being greater than 99.99% for all the three parameters for our experimental data.

**Table 2:** Results from the multiple linear regression test for the prediction of response variable storage modulus values based on the input variables of temperature, swelling ratio, and pH

Source	*p* value	Confidence of prediction (%)	Significance
Temperature	< 0.0001	99.9999	Highly Significant
Swelling Ratio	< 0.0001	99.9999	Highly Significant
pH	< 0.001	99.999	Highly Significant
Intercept	< 0.0001	99.9999	Highly Significant

Similarly, *p*-values lower than 0.02 were observed for the case of loss modulus as well, as documented in Table 3. Again, it was observed that the predictor variables could predict the loss modulus with a confidence of 99.99% or higher.

**Table 3:** Results from the multiple linear regression test for the prediction of response variable loss modulus values based on the input variables of temperature, swelling ratio, and pH

Source	*p* value	Confidence of prediction (%)	Significance
Temperature	< 0.0001	99.9999	Highly Significant
Swelling Ratio	< 0.0001	99.9999	Highly Significant
pH	< 0.001	99.999	Highly Significant
Intercept	< 0.0001	99.9999	Highly Significant

## Discussions

Several in vitro test methods have been proposed in previous studies to characterize the effects of the physiological conditions of the oral cavity on the viscoelastic behavior of denture adhesive cream. However, standard test models are generally lacking. This in vitro study investigated whether the parameters of pH, temperature, and swelling ratio influence the mechanical properties of denture adhesive cream. The data obtained in this study indicates that these parameters have a significant impact on mechanical behavior. This result is comparable to previous studies in the literature that have suspected and demonstrated a similar influence [17, 21]. .

**Fig. 6:**
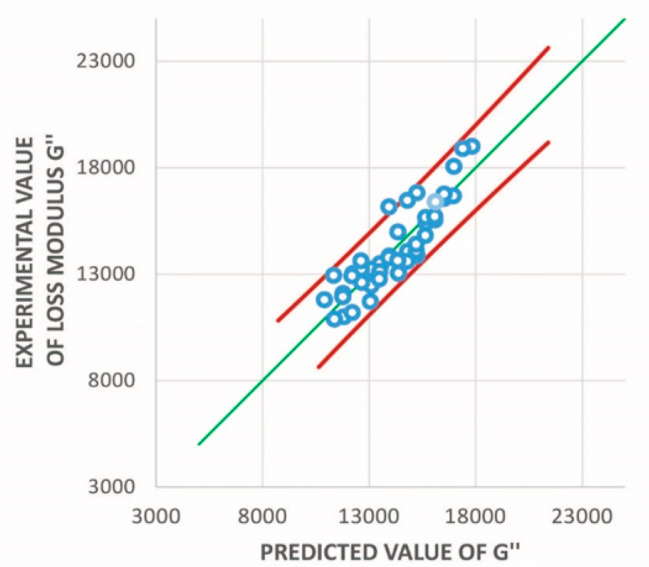
Plot showing the accuracy of predicting loss modulus values based on the curve fit in comparison to the experimentally determined values

The individual probability values presented in Tables 2 and 3 show that the null hypothesis can be rejected and that the three input variables of temperature, pH, and swelling ratio have a strong influence on the storage and loss modulus values. Figure 6 illustrates the predicted values of G´´ with respect to the corresponding experimentally determined values. The loss modulus plot has a regression parameter *R*-squared value of 0.88, which we consider to be a solid fit given the large variability and complexity of the test specimen. Additionally, the highest residuals were represented by only a few cases; excluding these values increased the *R*-squared value to around 0.96. The individual observations are depicted by the blue markers in Fig. 6 with the blue line indicating the fit function evaluated based on the multiple linear regression analysis. The red lines represent the 98% confidence level thresholds assumed in this study. From Fig. 6 it can be postulated that the fit function represents a good approximation for capturing the variation of the input variables.

Comparing the curves shown in Figs. 3 and 4 regarding dependence on the selected parameters, it is possible to estimate how they influence the mechanical properties of an adhesive cream. The influence of pH value can be seen in the figures, by comparing the horizontally adjacent graphs across the different pH values tested. Here, it is notable that the values for G´ and G´´ cover the same range of values for all three pH values, as well as for the swelling values of 60%, 100%, and 120%. As such, it can be concluded that the pH of saliva does not independently have a great influence on the storage modulus and loss modulus of the adhesive cream. The influence of swelling can be obtained by comparing the graphs located in each individual column. Here it can be seen that the curves shift lower in the diagram with increasing swelling, i.e. the values of G´ and G´´ become smaller with increasing swelling. This trend is observed similarly for pH values of 2, 7, and 10. With increased swelling, the values for the storage modulus and loss modulus decrease. That is, the damping properties of the adhesive cream decrease. In this study rheological testing was carried out once the adhesive attained specific levels of swelling when immersed in artificial saliva. As such, this is an idealized test scenario assuming that the volume of saliva or the flow rate of saliva is not changing. In reality, the salivary flow volume is constantly varying based on the secretion of saliva by the salivary glands as well as the flow rate constantly changing. The idealized scenario used in this study was implemented to simplify the variables involved and provide a qualitative understanding of the influence of swelling on the damping behavior of the adhesive. As such, it is argued that the swelling of the adhesive affects the damping behavior of the adhesive cream. Therefore, the degree of maximum swelling attained by denture adhesives in the oral cavity should be studied in greater detail in order to improve the comfort of denture wearers.

The influence of temperature and the fluctuations in the measured values must also be considered in each individual graph. In most cases, the curves are close together. At pH 2, the curves are widely spaced. The fluctuations in the values for G´ and G´´ are thus greater here than at pH 7 and pH 10. Generally, as the temperature increases, a decrease in viscosity is seen through the decreasing loss modulus values. This could be a result of long cross-linked polymer chains realigning with increasing temperature. Comparable results have been found in the works of Fallahi et al. (2018) and Gill et al. (2017) with similar investigations of the Poligrip denture adhesive and the role of physiological conditions [17, 21]. As reported in these studies, the influence of water content and temperature reduced the values of G´ and G´´; consequently, both have a significant bearing on adhesive mechanical properties [17, 21].

However, the three selected parameters cannot be considered individually in the oral cavity under physiological conditions, as they influence one another and all act simultaneously on the adhesive. In this study, the measurement ranges were extended or adapted closer to the physiologically relevant conditions. It must also be taken into account that saliva only has direct contact with a small part of the adhesive cream, namely at the denture margins. The selected frequency range describes the load a denture may experience in typical daily use. Thus, very low frequencies represent the resting state, i.e. an unloaded prosthetic, while higher frequencies simulate loads that can occur during occlusion. The chewing frequency can also vary as the mastication process progresses and as both the volume of the food and food particle size decrease. Higher chewing strokes are needed, and this increases linearly with the increasing volume of food. Based on the work of Po et al. (2011) the chewing frequency for the tested group ranged from 0.9 to 2.15 Hz, with a mean value of 1.58 Hz [23]. In the present work, the measurement spectrum was further expanded outside this range, from 0.01 to 10 Hz. The following movement sequences were hypothesized for the given frequencies: 0.01 Hz corresponds to the resting state, 1 Hz to slow chewing, and 10 Hz to fast chewing. The wider testing range was selected in order to encompass and take into account the entire spectrum of possible bite frequencies and to understand the material behavior from a mechanical and rheological standpoint. The study assumes an idealized bite force action, focusing only on the bite force application rather than a complete chewing cycle that would include both the opening and closing of the mandible in order to reduce the complexity of the variables involved.

With the selected pH range, the authors investigated physiological borderline cases which are only rarely exceeded or undershot in the oral cavity. For example, Loke et al. (2016) were able to determine pH values of 1.0 and 10.5 [24]. This is influenced by various factors such as salivary secretion and the content of the ingested food [16, 24]. Similarly, the temperature in the oral cavity is also influenced by various factors. The selected temperature range in this study covers more realistic temperatures compared to Fallahi et al. (2018) and Gill et al. (2017) [17, 21], who for instance have focused on larger increments in the temperature domain (i.e., 0, 37, and 60 °C) [17, 21]. Temperature within the oral cavity is a vital consideration and could lead to problems like denture burns. Furthermore, as the denture adhesive is present inside the oral cavity and experiencing both minor and major fluctuations in temperature because of both the dynamics of the oral cavity and also the incomplete contact of the adhesive to the oral cavity due to being covered by a chewable prosthesis with a minimum material thickness of 1.5 to 2 mm [25]. To study this very effect in greater detail and better understand the influence of more realistic and subtle changes in temperature, we have used smaller temperature intervals and investigated the adhesive at 5 °C steps between 17 and 52 °C.

If we now consider the collective influence of all the selected parameters on the denture adhesive, changes in the mechanical properties can be seen. Most striking are the larger temperature-dependent variations with increasing frequency, which occur for pH 2. This is not so noticeable for pH 7 and pH 10. Here the curves are more compactly spaced, and the fluctuations are smaller. However, the variation also increases with increasing frequency. This indicates that, on one hand, the temperature of an “acidic” food has more influence on the attenuating properties than that of a “neutral pH” food. On the other hand, it also depends on whether the patient is chewing, or the prosthetic is at rest. Thus, the temperature-dependent fluctuations increase with more intensive loads on the prosthetic. No uniform pattern is discernible to help determine at which temperature the best damping is achieved. This is not the same for the different pH values and threshold states. However, it is noticeable that especially at 47 and 52 °C the values of G´´ are smaller or even smallest, independent of the threshold rate. The crushing of a hot food, therefore, causes the pressure on the alveolar bone to be less well absorbed. In contrast, it is noticeable that for the resting state, i.e. a frequency of 0.01 Hz, the values at these high temperatures are often among the largest measured results. This means that the mechanical properties of denture adhesives are better at a high intraoral temperature without loading the prosthetic. However, that such a case occurs under physiological conditions is very unlikely, since such a large temperature change in the mouth can usually only be caused by food and hot beverages [19]. Therefore, 27 to 37 °C, i.e. temperatures close to the average of 34 °C [16], are of particular interest for consideration of the lower frequency range.

It can be seen from Fig. 5 that in the acidic and alkaline pH ranges a higher damping behavior is possible. For lower swelling values the loss modulus shows an almost constant behavior across the range of pH values at higher frequencies of loading. This variation with pH remains comparable even at lower frequencies, but the loss modulus is significantly lower at these levels. Again, the adhesive behavior for higher swelling values shows larger deviations with pH for the higher frequencies of loading.

**Fig. 7:**
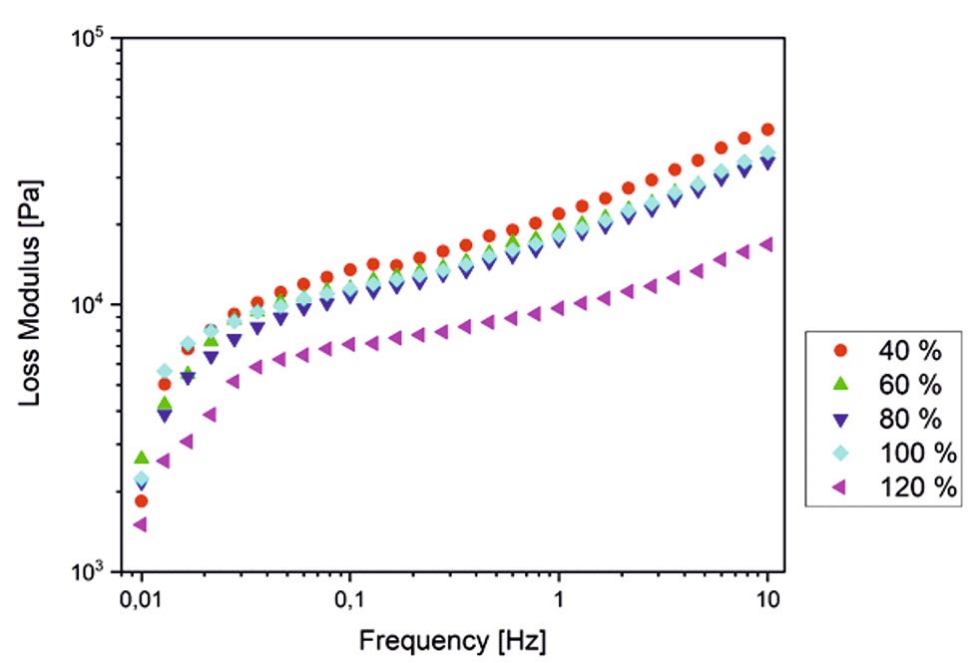
Effect of swelling on the loss modulus of the denture adhesive

The same is true for changes in pH, where the average value is 6.8 [15]. Here it is noticeable that G´´ for the average values in the lower frequency range usually assumes low or the lowest values, i.e. the worst attenuation prevails. If we look at the change in G´´ with increasing swelling under physiological conditions (Fig. 7), we see that the mechanical properties of the adhesive cream become less viscous, or the damping decreases. However, these properties increase during the process of mastication and dampen the pressure on the oral mucosa better during chewing than at rest. Particularly, when considered over numerous cycles of biting and chewing during the life cycle of a denture, this dampening is hypothesized to lower the cumulative stresses on the soft tissue and the underlying alveolar bone. Normally, the alveolar bone usually recedes when it is no longer physiologically loaded, for example when the teeth have been extracted. However, there is often so-called pressure atrophy, especially in wearers of full dentures, due to pressure transfer to the alveolar bone, which is caused by the fact that the blood supply in the bone is cut off by the pressure [26]. Akazawa and Sakurai (2002) showed that when loaded for more than 20 s, these pressures were associated with a significant decrease in blood flow and a continuous clenching could delay recovery of this flow [27]. A component of this generated pressure is also expected to result in the elastic deformation of the denture adhesive itself, which then fills the gaps or uneven regions in the denture-mucosa interface due to pressure equalization. However, in this study the investigations were carried out using a parallel plate rheometer on a layer of the denture adhesive having a specified thickness and not with the actual dental prosthesis which can potentially have uneven contact regions. Hence, the component of the pressure responsible for the denture adhesive’s elastic deformation could not be assessed in this study and the lowering of the contact pressure due to the denture adhesive, as exhibited from the results, was primarily attributed to its damping behavior. As such, the damping of local pressure on the soft tissue and the alveolar bone over the life cycle of the denture has important clinical relevance for dental professionals, as it can reduce alveolar bone degradation [28] and thus help to maintain or fabricate a well-fitting prosthesis.

In this study, the authors investigated the extent to which the mechanical properties of denture adhesives change under the physiological conditions of the oral cavity. The limitations of this in vitro study include the individual salivary composition [29] of each patient, the varying degrees of swelling of the product depending on how much liquid is in direct contact with the adhesive, and the real temperature that the adhesive can reach when under the denture, which has a minimum layer thickness of 1.5-2.0 mm [25]. The influence of the adhesive thickness and the implications of its variation on the stress state of the dental structures and oral mucosa was not considered in this study in order to simplify the experimental investigations. Furthermore, the kinematics of the denture-oral mucosa contact due to the presence of the denture adhesive when the bite force is applied and released is also beyond the scope of this study, as the focus was primarily on the rheological investigation and interpretation of these results. As such, the influence of the tangential shearing component forces that are generated because of an applied bite force on this interface could not be analyzed in this study. Moreover, the adhesive introduces different contact mechanical conditions on the film than saliva and its swelling helps to compensate for inaccuracies of the contour due to any manufacturing errors or changes in the shape of the foundation.

In this context, the study was able to highlight that the state of the oral cavity is a very dynamic and complex environment, and that the performance of a denture adhesive in this environment is influenced by several of these variables. Amongst the physiological conditions of the oral cavity, this study demonstrates that the temperature, pH, and swelling due to the influence of saliva significantly influence the mechanical response of the denture adhesive. The data recorded in this study can be used as inputs for factoring in the influence of these physiological variables for developing a complex viscoelastic material law based on approaches like Prony series approximation [30].

## Conclusions

The study highlights that for the same bite load, an adhesive at different temperatures behaves differently, and further, for the same temperature the adhesive shows varying levels of damping based on the degree of swelling. It was observed that when considered collectively their role could potentially be a strong determinant in better characterizing these adhesives. The data generated from this research was also one of the several inputs towards developing a multi-parameter model of the mechanical behavior of denture adhesive creams. Such a model could be used for numerically simulating denture adhesive behavior and its response to various physiological and mechanical forces acting within the oral cavity.

## Data Availability

The datasets used and/or analyzed during the current study are available from the corresponding author on reasonable request.

## References

[1] Yohei Okazaki Y, Abe K, Dainobu S, Iwaguro (2021). Ryoji Kato, an Kazuhiro Tsuga. A web-based survey of denture adhesive use among denture wearers 40 years of age and older. J Oral Sci.

[2] Psillakis JJ, Wright RF, Grbic JT, Lamster IB (2004). In practice evaluation of a denture Adhesive using a gnathometer. J Prosthodont Wiley.

[3] Lemos CAA, Fonte Porto Carreiro A da, Rosa CDDRD, Gomes JML, de Oliveira Limirio JPJ, Mendonça G et al. Does the use of an adhesive improve conventional complete dentures? A systematic review of randomized controlled trials. The Journal of Prosthetic Dentistry. Elsevier BV; 2021. 10.1016/j.prosdent.2020.11.041 33551134

[4] Kapur KK (1967). A clinical evaluation of denture adhesives. J Prosthet Dentistry Elsevier BV.

[5] Adisman IK (1989). The use of denture adhesive as an aid to denture treatment. J Prosthet Dentistry Elsevier BV.

[6] Munoz CA, Gendreau L, Shanga G, Magnuszewski T, Fernandez P, Durocher J (2011). A clinical study to Evaluate Denture Adhesive Use in Well-Fitting dentures. J Prosthodont Wiley.

[7] de Oliveira Junior NM, Rodriguez LS, Marin DOM, Paleari AG, Pero AC, Compagnoni MA (2014). Masticatory performance of complete denture wearers after using two adhesives: a crossover randomized clinical trial. J Prosthet Dentistry Elsevier BV.

[8] Gonçalves T, Viu F, Gonçalves L, Garcia R (2014). Denture adhesives improve mastication in denture wearers. Int J Prosthodont Quintessence Publishing.

[9] Kalra P, Nadiger R, Shah FK (2012). An investigation into the effect of denture adhesives on incisal bite force of complete denture wearers using pressure transducers - a clinical study. J Adv Prosthodont Korean Acad Prosthodont.

[10] Kano H, Kurogi T, Shimizu T, Nishimura M, Murata H (2012). Viscosity and adhesion strength of cream-type denture adhesives and mouth moisturizers. Dent Mater J Japanese Soc Dent Mater Devices.

[11] Han J-M, Hong G, Dilinuer M, Lin H, Zheng G, Wang X-Z (2014). The adhesive strength and initial viscosity of denture adhesives. Acta Odontol Scand Informa UK Ltd.

[12] Lenz M, Greess H, Baum U, Dobritz M, Kersting-Sommerhoff B (2000). Oropharynx, oral cavity, floor of the mouth: CT and MRI. Eur J Radiol Elsevier BV.

[13] Souza LR, Oliveira MVM, Basile JR, Souza LN, Souza ACR, Haikal DS et al. Anatomical and physiopathological aspects of oral cavity and Oropharynx Components related to Oropharyngeal Dysphagia. Seminars in Dysphagia. InTech; 2015.

[14] Nicolas E, Veyrune J, Lassauzay CA, Six-Month (2010). Assessment of oral health-related quality of life of complete denture wearers using denture adhesive: a pilot study. J Prosthodont Wiley.

[15] Loke C, Lee J, Sander S, Mei L, Farella M (2016). Factors affecting intra-oral pH - a review. J Oral Rehabilitation Wiley.

[16] Choi JE, Lyons KM, Kieser JA, Waddell NJ. Diurnal variation of intraoral pH and temperature. Volume 3. BDJ Open. Springer Science and Business Media LLC; 2017. 10.1038/bdjopen.2017.15 PMC5842828 29607085

[17] Fallahi A, Khadivi N, Roohpour N, Middleton AM, Kazemzadeh-Narbat M, Annabi N (2018). Characterization, mechanistic analysis and improving the properties of denture adhesives. Dent Mater Elsevier BV.

[18] Moore R. Intra-oral temperature variation over 24 hours. The European Journal of Orthodontics. Volume 21. Oxford University Press (OUP); 1999. pp. 249–61. 10.1093/ejo/21.3.249 10407534

[19] Barclay CW, Spence D, Laird WRE (2005). Intra-oral temperatures during function. J Oral Rehabilitation Wiley.

[20] Turner M, Jahangiri L, Ship JA (2008). Hyposalivation, xerostomia and the complete denture. J Am Dent Association Elsevier BV.

[21] Gill SK, Roohpour N, Topham PD, Tighe BJ (2017). Tunable denture adhesives using biomimetic principles for enhanced tissue adhesion in moist environments. Acta Biomater Elsevier BV.

[22] Pratten J, Smith AW, Wilson M. Response of single species biofilms and microcosm dental plaques to pulsing with chlorhexidine. Journal of Antimicrobial Chemotherapy. Volume 42. Oxford University Press (OUP); 1998. pp. 453–9. 10.1093/jac/42.4.453 9818743

[23] Po JMC, Kieser JA, Gallo LM, Tésenyi AJ, Herbison P, Farella M. Time-frequency analysis of chewing activity in the natural environment. Volume 90. SAGE; 2011. pp. 1206–10. 10. 10.1177/0022034511416669 21810620

[24] Loke C, Lee J, Sander S, Mei L, Farella M (2016). Factors affecting intra-oral ph–a review. J Rehabil.

[25] Nawarah Alaseef S, Albasarah HA, Abdulghani, Fahad A, Al-Harbi MM, Gad S, Akhtar SQ, Khan (2022). Ijlal Shahrukh Ateeq, and Faisal Dal Qarni. Cad-cam fabricated denture base resins: in vitro investigation of the minimum acceptable denture base thickness. J Prosthodont.

[26] Maruo Y, Nishigawa G, Irie M, Oka M, Hara T, Suzuki K, Minagi S (2010). Stress distribution prevents ischaemia and bone resorption in residual ridge. Arch Oral Biol.

[27] Akazawa H, Sakurai K (2002). Changes of blood flow in the mucosa underlying a mandibular denture following pressure assumed as a result of light clenching. J Rehabil.

[28] Gunnar E, Carlsson (2004). Responses of jawbone to pressure. Gerodontology.

[29] Neyraud E, Palicki O, Schwartz C, Nicklaus S, Feron G (2012). Variability of human saliva composition: possible relationships with fat perception and liking. Arch Oral Biol.

[30] Ramakrishnan AN, Röhrle O, Ludtka C, Varghese R, Koehler J, Kiesow A, et al. Finite element evaluation of the Effect of Adhesive creams on the stress state of dentures and oral mucosa. In: Rong Q, editor. Applied Bionics and Biomechanics. Volume 2021. Hindawi Limited; 2021. pp. 1–9. 10.1155/2021/5533770 PMC8128609 34046080

